# Cataract Surgery with a Refractive Corneal Inlay in Place

**DOI:** 10.1155/2015/230801

**Published:** 2015-06-11

**Authors:** N. R. Stojanovic, S. I. Panagopoulou, I. G. Pallikaris

**Affiliations:** Institute of Vision and Optics (IVO), Medical School, University of Crete, Heraklion, 70013 Crete, Greece

## Abstract

*Purpose*. To present a case of cataract surgery performed in a patient with a refractive corneal inlay in place. *Methods*. A 48-year-old female patient presented to our institute with bilateral cataract. The patient had undergone refractive corneal inlay implantation three years ago in her right, nondominant eye for presbyopia correction. Biometry and intraocular lens (IOL) power calculation were performed without removing the inlay. Phacoemulsification and IOL insertion were carried out in both eyes in a usual manner. *Results*. On day one postoperatively, the patient achieved binocular uncorrected distance visual acuity 20/20 and uncorrected near visual acuity J1. The vision remained stable during the one-year follow-up period. *Conclusion*. Cataract surgery was performed in a standard manner in a patient with Presbia Microlens corneal inlay in place. Visual outcomes for both near and distance vision were satisfactory.

## 1. Introduction

In recent years, several corneal procedures have been proposed for presbyopia treatment including monovision laser in situ keratomileusis (LASIK), photorefractive keratectomy (PRK), conductive keratoplasty (CK), presbyopic LASIK (presbyLASIK), and, more recently, the IntraCor technique and the corneal inlay [1]. The biggest advantage of corneal inlays is the fact that they are additive and do not remove tissue, and they therefore preserve future options for any kind of presbyopia correction as discussed by Lindstrom et al. [2]. Corneal inlays are placed under stromal flaps or inside stromal pockets made by microkeratomes or femtosecond lasers. Different inlay models are reported to use different mechanisms to compensate for accommodation loss, such as positive refractive power, change of anterior corneal curvature, or increase of the depth of field by fixed small aperture [3].

The satisfactory outcomes regarding efficacy and patients' satisfaction after the inlay implantation for presbyopia could be changed by cataract development, due to the normal aging process, resulting in vision deterioration. Given that the number of presbyopic patients with the corneal inlays is increasing, it is important to address some issues regarding cataract surgery in these patients.

We describe our experience of cataract surgery in a patient with the Presbia Microlens corneal inlay in place.

## 2. Case Presentation

A 48-year-old female patient presented to our institute with a history of blurred vision for the last six months. The patient had undergone refractive corneal inlay implantation three years ago in her right, nondominant eye for presbyopia correction. At presentation, the patient had uncorrected distance visual acuity (UDVA) 20/40 in the right eye, 20/32 in the left eye, and 20/32 binocularly. Uncorrected near visual acuity (UNVA) was J1 in the right eye, J3 in the left eye, and J1 binocularly. The patient achieved corrected distance visual acuity (CDVA) 20/32 with refraction +1.00 − 1.25 × 180 in the nondominant and 20/25 with +0.75 − 0.25 × 165 in the dominant eye. Slit-lamp examination revealed nuclear sclerosis and posterior subcapsular cataract (NC3 and P3 according to the Lens Opacities Classification System III (LOCS III)) in both eyes [4]. The remaining anterior and posterior segment findings were unremarkable.

After we discussed all options, the patient opted for bilateral cataract surgery without removal of the corneal inlay in order to improve her far and preserve near vision and spectacle independence. The written informed consent was obtained from the patient.

Routine preoperative evaluation for cataract surgery was performed. Biometry was performed with IOL Master (Carl Zeiss Meditec, Jena, Germany) in a usual manner. The surgeon opted for intraocular lens (IOL) power calculated with SRK-T formula for a one-piece monofocal intraocular lens (AcrySof IQ SN60WF, Alcon), targeting emmetropia.

**Figure 1:**
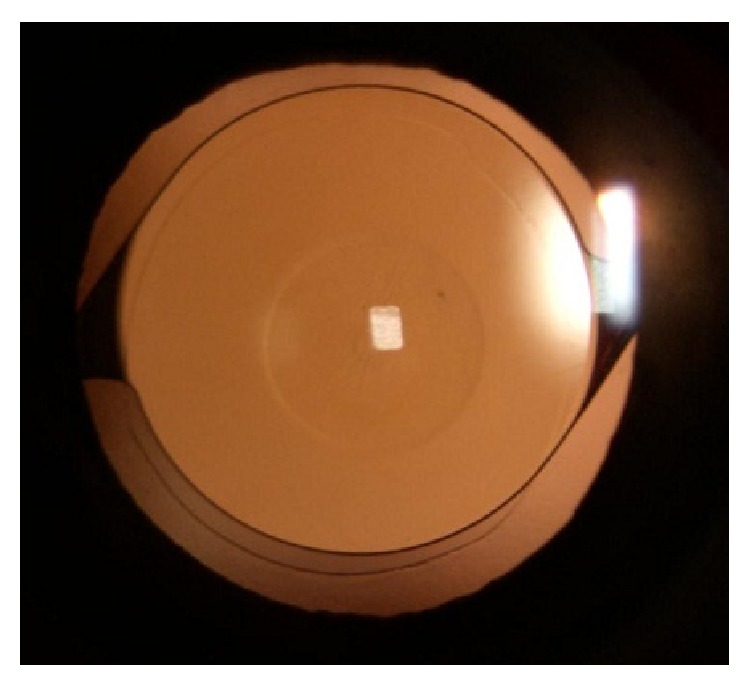
A slit-lamp retroillumination photograph of the Presbia Microlens and intraocular lens.

The bilateral cataract extraction with phacoemulsification and posterior chamber IOL implantation was carried out first in the nondominant and two days later in the dominant eye, which is the surgeon's usual approach. The surgeries were performed under sterile conditions with topical anaesthesia. A clear corneal incision of 2.8 mm was made, and an anterior curvilinear capsulorhexis of 5.5 mm was performed. Phacoemulsification was performed using the Infiniti Vision System (Alcon Laboratories, Inc., Fort Worth, Texas), with thorough cortical removing and meticulous cleaning of the posterior capsule and anterior capsular leaflets. After phacoemulsification and lens removal, the IOLs (AcrySof IQ SN60WF OD +21.5D and OS +21.5D) were implanted into the capsular bag using the standard injector device. The surgery was uneventful in both eyes (Figure 1).

Postoperative topical therapy included topical antibiotic-steroid drops (tobramycin/dexamethasone, Tobradex; Alcon Laboratories, Inc., Ft Worth, Texas) four times a day for 4 weeks with a weekly tapering regimen.

**Figure 2:**
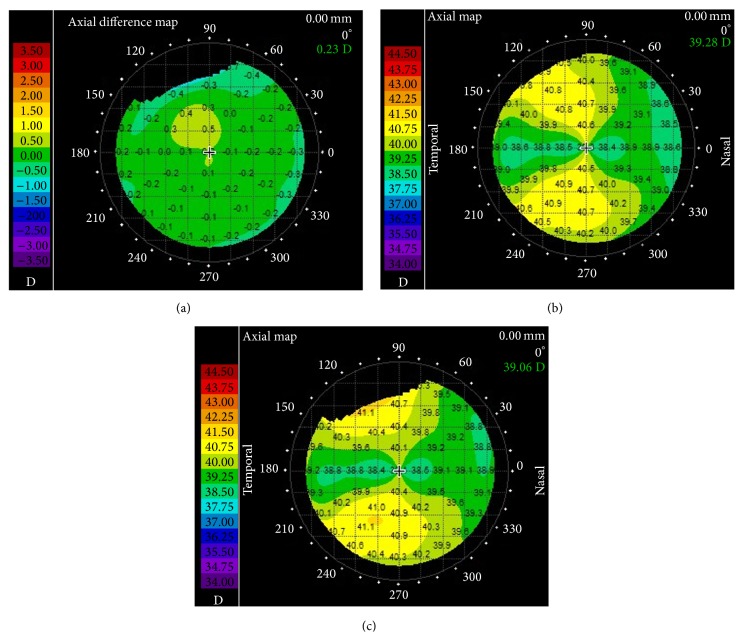
Corneal topography maps preoperatively and one year after the cataract surgery (the map on (b) is preoperative and the map on (c) is one-year postoperative axial map; the map on (a) is the pre- and postoperative axial differential map).

On postoperative day one, the patient had UDVA 20/40 in her right (OD) and 20/20 in her left eye (OS), 20/20 binocularly and UNVA OD J1 and OS J3, and J1 binocularly. One year postoperatively, the patient yielded UDVA of 20/32 in the nondominant and 20/20 in the dominant eye and CDVA of 20/20 bilaterally with manifest refraction +0.25 − 1.25 × 170 and +0.50, respectively. The patient had binocular UDVA 20/20 and UNVA J1. Topography findings did not show significant change before and after cataract surgery (Figure 2).

No complications were recorded on any of the follow-up visits. The patient was happy with the final visual outcome and remained spectacle-free.

## 3. Discussion

Intrastromal corneal inlays are a new modality for presbyopia correction. The Presbia Microlens (Presbia, Amsterdam, Netherlands) is a transparent, hydrophilic disc with 3 mm diameter and approximately 15 *μ*m edge thickness. The central 1.6 mm diameter of the disc is plano in power and the peripheral zone has the additional positive power. The lens has a bifocal optical system which acts as modified monovision and is inserted into the intrastromal corneal pocket made by femtosecond laser in the nondominant eye. Our previous study showed that refractive corneal inlay is safe and effective method for presbyopia correction [5]. However, some patients may eventually develop cataract and require cataract surgery. At present, there are several available options, including cataract surgery with the inlay in place, inlay removal followed by cataract surgery and subsequent inlay reimplantation, and inlay removal followed by cataract surgery with implantation of an accommodative or multifocal intraocular lens. However, if a patient does not wish to remove the refractive inlay, then monofocal intraocular lens should be used. When choosing the IOL power, emmetropia should be targeted, given that the Presbia Microlens is a refractive lens (with positive refractive power).

The major concerns regarding cataract surgery with a corneal inlay in place are the accuracy of preoperative evaluation and biometry readings, technical aspects of the surgery, and visual outcomes. In our case, the preoperative evaluation was performed in a standard manner. The slit-lamp evaluation of anterior and posterior segment was not affected by the inlay due to its transparency. Fundus and iridocorneal angle examination with Goldmann three-mirror contact lens have been performed without any difficulty.

The results of manifest refraction one year after surgery in both eyes suggest that biometry readings and IOL power calculations were reasonably accurate. Biometry findings taken from IOL Master and calculated refraction are presented in Table 1. Regarding the formulas, it would seem that both SRK/T and Hoffer Q provided satisfying results, but one case is not sufficient to establish validity of either formula in patients with Presbia Microlens.

Technical aspects of the surgical procedure were not in the least affected by Microlens. The transparent inlay provides excellent visibility through the operating microscope and allows all the usual surgical manipulations.

In conclusion, in our case, phacoemulsification and intraocular lens implantation were performed in a patient with Presbia Microlens corneal inlay without any modification or additional surgical manoeuvre. Visual outcomes for both near and distance vision were satisfactory. The inlay does not appear to have had significant effect on biometry or IOL power calculation. However, larger studies are needed for drawing definite conclusions regarding safety and visual outcome of cataract surgery with the refractive corneal inlay in place as well as establishing the appropriate formula for calculation of intraocular lens power.

## Figures and Tables

**Table 1:** Biometry readings and target refractions for +21.5 D intraocular lens calculated with three different formulas and manifest refraction one year after surgery.

	AL (mm)	*K*1 (D)	*K*2 (D)	ACD (mm)	SRK/T Ref (D)	Hoffer Q Ref (D)	Haigis Ref (D)	Manifest refraction after cataract surgery (D)
OD	24.67	39.79	41.31	3.48	−0.06	0.5	0.76	+0.25 − 1.25 × 170
OS	24.54	39.99	40.71	3.40	−0.03	0.5	0.70	+0.50

AL, axial length; *K*1 and *K*2, keratometry readings; ACD, anterior chamber depth; Ref (D), calculated refraction.
